# Statins in Breast Cancer Therapy: Mechanistic Insights and Emerging Evidence

**DOI:** 10.1002/cai2.70040

**Published:** 2026-01-16

**Authors:** Rohina Alim, H. M. Kasuni Akalanka

**Affiliations:** ^1^ Rural Health Research Institute Charles Sturt University Orange New South Wales Australia

**Keywords:** anticancer therapy, apoptosis, breast cancer, cell proliferation, drug repurposing, mevalonate pathway, simvastatin, statins, triple‐negative breast cancer

## Abstract

Breast cancer (BC) remains the most frequently diagnosed malignancy worldwide, with an estimated 2.3 million new cases and approximately 685,000 deaths reported in 2020. Forecasts suggest a substantial rise in global incidence, with new annual cases projected to reach 3.2 million by 2050, representing a 39% increase. Additionally, BC is expected to account for approximately 7.7% of the anticipated $25.2 trillion global economic burden associated with cancer by 2050. These trends underscore an urgent need for affordable, widely accessible and effective therapeutic strategies, particularly in low‐ and middle‐income countries. Statins, commonly prescribed for the treatment of hypercholesterolaemia via inhibition of 3‐hydroxy‐3‐methylglutaryl‐coenzyme A (HMG‐CoA) reductase, have garnered increasing interest for their potential anticancer properties. This review focuses on the mechanistic underpinnings and therapeutic implications of statin use, particularly simvastatin, in the context of BC. Statins exert their primary effect through inhibition of the mevalonate pathway, which is crucial for cholesterol and isoprenoid biosynthesis. Disruption of this pathway impairs the prenylation of key signalling proteins, including members of the Ras and Rho GTPase families, which are essential for cancer cell proliferation, survival and metastasis. Preclinical evidence has demonstrated that simvastatin can induce tumour cell apoptosis, arrest cell‐cycle progression and inhibit oncogenic signalling pathways. These effects have been particularly pronounced in hormone receptor‐negative and triple‐negative breast cancer (TNBC) subtypes, which are often associated with poor prognosis and limited treatment options. Epidemiological and observational studies further support a potential association between statin use and reduced BC recurrence and mortality. Nevertheless, robust evidence from randomised controlled trials remains limited, and further investigation is required to establish causality and define optimal therapeutic regimens. Given their well‐established safety profile, global accessibility and pleiotropic effects, statins, especially simvastatin, represent a promising class of repurposed drugs in the adjuvant treatment of BC. This review synthesises evidence from the past two decades, highlighting the need for continued clinical research to validate and optimise the use of statins as adjunctive agents in BC therapy.

## Graphical abstract

Statins, particularly simvastatin, demonstrate potential as adjuvant agents in breast cancer treatment by disrupting tumour cell signalling and survival pathways. This review highlights emerging evidence supporting their repurposing for improved outcomes, especially in aggressive breast cancer subtypes.

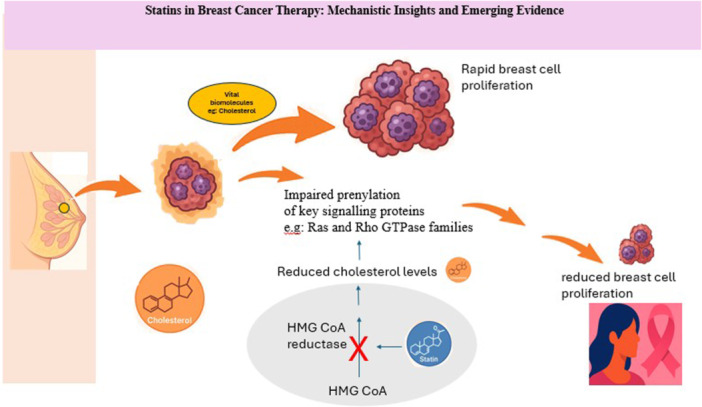

**Abbreviations**
BC: breast cancer
CHOP: C/EBP homologous protein
CoQ10: coenzyme Q10
DR5: death receptor 5
ER: estrogen receptors
FASN: fatty acid synthase
FPP: farnesyl pyrophosphate
HER2: human epidermal growth factor receptor 2
HMG‐CoA: 3‐hydroxy‐3‐methylglutaryl coenzyme A
IC50: half‐maximal inhibitory concentration
IDC: invasive ductal carcinoma
IDFS: invasive disease‐free survival
ILC: invasive lobular carcinoma
JNK: c‐Jun N‐terminal kinase
LDL‐C: low‐density lipoprotein cholesterol
LMICs: low‐ and middle‐income countries
MASTER Trial: MAmmary cancer STatins in ER positive breast cancer
MAPK: mitogen‐activated protein kinase
MCF‐7: hormone receptor‐positive breast cancer
MCM7: minichromosome maintenance complex component 7
MDA‐MB‐231: triple‐negative subtype cell line
OATPs: organic anion‐transporting polypeptides
OS: overall survival
PARP: poly (ADP‐ribose) polymerase
PI3K: phosphoinositide 3‐kinase
PON1: paraxonase 1
PR: progesterone receptors
Rb: retinoblastoma protein
SASP: senescence‐associated secretory phenotype
TNBC: triple‐negative breast cancer
WHO: World Health Organization
YAP/TEAD: Yes‐associated protein/TEA domain transcription factor

## 1 Introduction

Breast cancer (BC) is a heterogeneous group of malignancies that most commonly arises from the epithelial cells of the breast ducts and lobules and remains a significant global health concern. Although it predominantly affects women, it also occurs in men and transgender individuals [1, 2]. BC is currently the most diagnosed cancer worldwide, with an estimated 2.3 million new cases and 685,000 deaths reported in 2020 [3]. The World Health Organization (WHO) anticipates a substantial increase in the global burden of BC, projecting approximately 3.2 million new cases and 1.1 million associated deaths by 2050. These projections underscore the pressing need for robust prevention, early detection and treatment interventions to mitigate the anticipated rise [4, 5]. This projected rise represents a 39% increase from the current 2.3 million annual cases. BC accounts for 7.7% of the estimated $25.2 trillion global cancer‐related economic burden from 2020 to 2050 [6], with the impact disproportionately affecting low‐ and middle‐income countries (LMICs) due to higher mortality rates and limited access to timely diagnosis and effective treatment [7]. Notably, annual mortality rates in regions such as South Asia and Sub‐Saharan Africa are nearly twice those observed in high‐income countries, contributing to a growing welfare loss relative to GDP [7].

The development of BC is influenced by a combination of inherited susceptibility and modifiable environmental and lifestyle factors. Advancing age, hormonal exposure, obesity, and prior radiation treatment are well‐established contributors to risk [8, 9]. These factors can lead to molecular alterations within the epithelial cells of the breast, particularly in the ductal or lobular structures, initiating uncontrolled proliferation and, in some cases, metastatic spread [8]. Histologically, most BC cases arise in the milk ducts (invasive ductal carcinoma, IDC), while a smaller proportion originates in the lobules (invasive lobular carcinoma, [ILC]) [10].

The role of altered lipid metabolism is identified as a key contributor to BC progression. Malignant cells frequently exhibit upregulation of de novo fatty acid synthesis, mediated by enzymes such as fatty acid synthase (FASN) [11, 12]. This metabolic reprogramming supports the biosynthesis of membrane lipids and lipid‐derived signalling molecules, thereby promoting tumour cell growth, survival and invasiveness [12]. Moreover, dysregulated lipid metabolism fosters oxidative stress, immunosuppressive changes within the tumour microenvironment and resistance to conventional chemotherapeutic agents, with pronounced effects in aggressive subtypes such as triple‐negative breast cancer (TNBC) [13].

**Figure 1:**
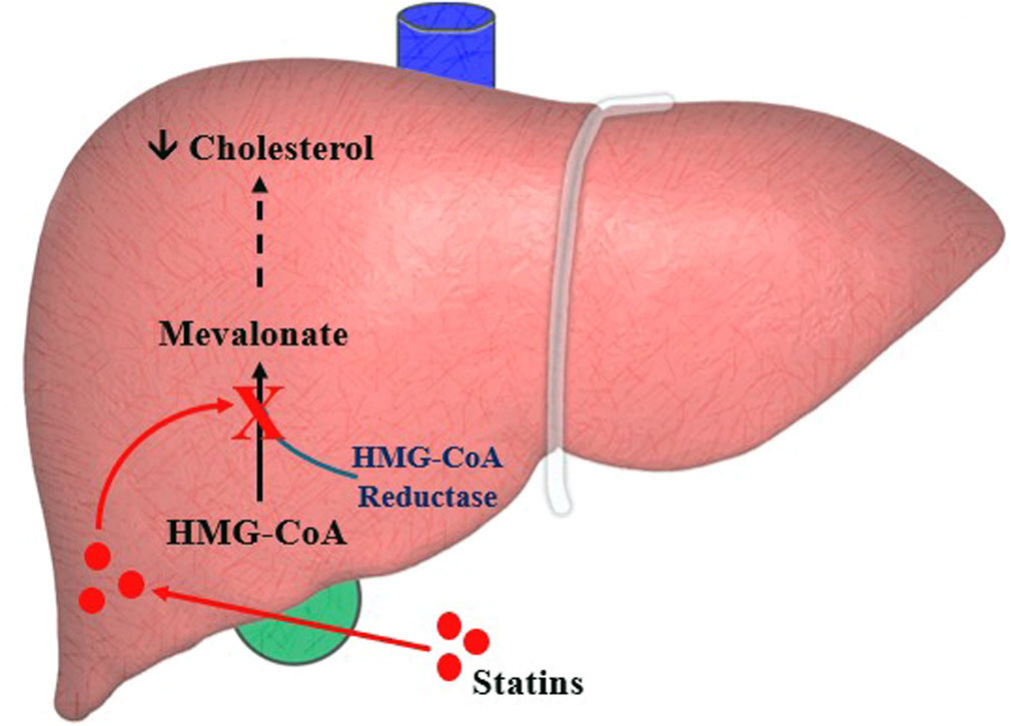
Schematic representation of the mode of action of simvastatin. The illustration was created using Microsoft PowerPoint, with information sourced from [14, 15].

Statins (Figure 1) function as HMG‐CoA (3‐hydroxy‐3‐methylglutaryl coenzyme A) reductase inhibitors that block the rate‐limiting step in cholesterol biosynthesis by inhibiting the mevalonate pathway [16, 17]. HMG‐CoA reductase is a key enzyme that catalyses the conversion of HMG‐CoA to mevalonate, a crucial precursor in the synthesis of cholesterol and other isoprenoids [16, 17]. By targeting this enzyme, statins reduce the production of cholesterol and isoprenoid intermediates essential for protein prenylation, a process critical for cell signalling and proliferation [18, 19].

Statin therapy is also known to reduce endogenous coenzyme Q10 (CoQ10) levels through the inhibition of the mevalonate pathway [20]. Experimental studies have shown that statins can significantly decrease CoQ10 concentrations in various tissues. For example, Tavintharan et al. demonstrated that simvastatin treatment reduced CoQ10 levels and impaired mitochondrial respiratory chain activity in human hepatoma cells, linking statin‐induced CoQ10 deficiency to mitochondrial dysfunction [21]. Similarly, experimental evidence demonstrates that atorvastatin can lead to reductions in cellular CoQ10 levels and impair mitochondrial function across several cell types [22]. For instance, Urbano et al. showed that atorvastatin reduced expression of CoQ10B protein, caused oxidative stress, and impaired mitochondrial activity in INS‐1 pancreatic β‐cells, with mevalonate reversing these mitochondrial defects [22]. Additionally, Zhang et al. found that atorvastatin induced mitochondrial dysfunction and membrane potential loss, resulting in cell death through ferroptosis mechanisms in human cardiomyocyte and muscle cell models [23]. In cancer cells, McGregor et al. reported that statin‐mediated CoQ depletion leads to impaired oxidative phosphorylation, elevated reactive oxygen species and a compensatory metabolic shift, directly linking statin‐induced mitochondrial dysfunction via CoQ10 loss to cytotoxic effects in cancer cells [24]. These findings support the hypothesis that statin‐induced mitochondrial dysfunction, driven by decreased CoQ10, may contribute to their anticancer activity.

While statins are primarily used to manage hypercholesterolaemia, emerging evidence suggests they may have therapeutic potential in BC by disrupting tumour cell growth and survival [25, 26]. Clinical and preclinical studies indicate statins can impair cancer cell signalling, modulate the lipid‐rich tumour microenvironment and enhance the effectiveness of cytotoxic chemotherapy [18, 26, 27]. Observational data show associations between statin use and improved BC specific survival, particularly in hormone receptor‐positive subtypes, and ongoing trials are investigating their role as adjunctive agents to standard therapies [25, 26].

In this review, we explore the potential role of statins in BC management, focusing on different types of statins, with simvastatin showing greater effectiveness, its mode of action in BC and its clinical applications.

## 2 Physicochemical Classification of Statins

Statins are commonly classified based on their physicochemical properties, particularly lipophilicity and hydrophilicity, which significantly influence their pharmacokinetic behaviour, tissue distribution and potential for adverse effects [28]. This classification divides statins into two primary groups: lipophilic and hydrophilic statins, each exhibiting distinct absorption and cellular uptake characteristics [28].

**Figure 2:**
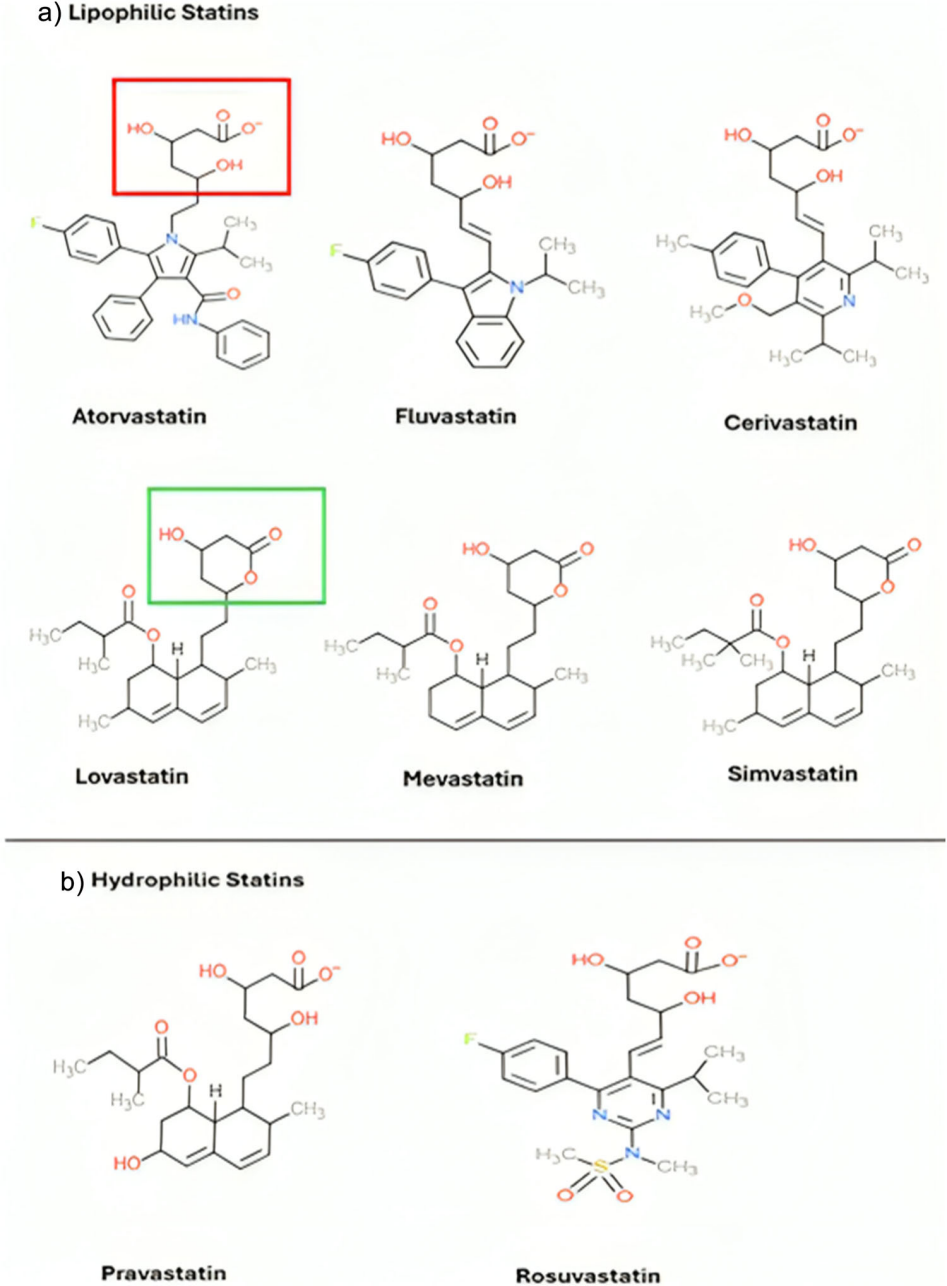
Chemical structures of statins. (a) Chemical structures of lipophilic statins highlighting the common pharmacophore site, with the open‐ring form indicated in the red box and the closed‐ring form in the green box. (b) Chemical structures of hydrophilic statins, illustrated using Demos > 2D Sketcher (ChemDoodle); structures adapted from Espano et al., *Lipophilic statins inhibit Zika virus production in Vero cells, Scientific Reports*, licensed under CC BY 4.0 https://www.nature.com/articles/s41598-019-47956-1, with modifications [29].

Lipophilic statins that include atorvastatin, fluvastatin, cerivastatin, lovastatin, mevastatin and simvastatin (Figure 2a) are highly lipid‐soluble and can passively diffuse across cellular membranes, facilitating their distribution into a variety of tissues beyond the liver [29, 30].

For example, Clemente et al. [31] and Gaber [32] used positron emission tomography (PET) imaging and biodistribution studies in rats to demonstrate that atorvastatin, a lipophilic statin, exhibits the capacity for extensive tissue distribution due to its high membrane permeability, with the majority of uptake occurring in the liver but measurable distribution in extrahepatic tissues as well. This widespread systemic distribution may enhance therapeutic effects in extrahepatic tissues but is also associated with an increased risk of off‐target adverse effects, such as myopathy and potential drug–drug interactions [33].

In contrast, hydrophilic statins (Figure 2b) such as pravastatin and rosuvastatin are more water‐soluble and rely on active transport mechanisms, particularly organic anion‐transporting polypeptides (OATPs), for hepatic uptake [33, 34]. Kitamura et al. [34] showed that rosuvastatin uptake by hepatocytes is mediated by OATP1B1, contributing to its high hepatoselectivity and limited penetration into peripheral tissues. As a result, hydrophilic statins predominantly exert their cholesterol‐lowering effects within the liver and are generally associated with a reduced incidence of systemic side effects compared with their lipophilic counterparts [33].

Extending beyond their pharmacokinetic distinctions, Liu et al. [35] conducted a meta‐analysis examining the association between statin type, exposure duration and BC prognosis across multiple clinical cohorts. The study found that lipophilic statins, such as simvastatin and atorvastatin, were associated with improved BC‐specific survival and overall outcomes, whereas hydrophilic statins demonstrated weaker or nonsignificant associations. These findings suggest that the enhanced membrane permeability and intracellular access of lipophilic statins may confer additional antitumour effects beyond cholesterol lowering [35]. This analysis strengthens the evidence that physicochemical differences among statin classes translate into distinct biological and clinical impacts, emphasising the importance of considering statin type in both cardiovascular and oncology contexts [35].

Building on these pharmacological and clinical distinctions, at standard therapeutic doses of 20–40 mg per day, statins achieve plasma concentrations in the low nanomolar range, sufficient to inhibit hepatic HMG‐CoA reductase, the rate‐limiting enzyme in cholesterol biosynthesis, with an in vitro IC₅₀ of approximately 10–20 nM [36]. This potent inhibition leads to significant reductions in cholesterol biosynthesis and LDL cholesterol; for example, in the Scandinavian Simvastatin Survival Study (4S), 20–40 mg daily resulted in a 30% reduction in all‐cause mortality (relative risk 0.70, 95% CI: 0.58–0.85, *p* = 0.0003) [37].

Statins exist in two structural forms: an open‐ring (hydroxy acid, Figure 2a, red box) configuration, which is the pharmacologically active state, and a closed‐ring (lactone, Figure 2a, green box) form, which is inactive and must undergo in vivo hydrolysis to become active [38, 39]. This distinction underpins differences in pharmacokinetics, with lactone‐form statins (e.g., lovastatin, simvastatin) functioning as prodrugs that require enzymatic activation in the liver, while those administered in the open‐ring form (e.g., atorvastatin and rosuvastatin) are immediately active [39, 40]. The lactone rings of statins can be hydrolysed by paraoxonases, enzymes present in many cell types, which enables activation of statin lactones such as simvastatin and lovastatin even in non‐hepatic in vitro systems. Paraoxonase 1 (PON1), in particular, has been shown to hydrolyse statin lactones, converting them into their pharmacologically active hydroxyacid forms [41]. This enzymatic activity explains how simvastatin and lovastatin can exhibit biological effects in cell culture studies lacking liver metabolism. Khersonsky and Tawfik demonstrated that PON1 exhibits significant lactonase activity towards a range of substrates, including hydrophobic lactones similar to statin lactones, suggesting that lactone hydrolysis is a native function of this enzyme [42]. Additionally, Riedmaier et al. provide evidence that both PON1 and the related enzyme PON3 contribute to atorvastatin‐lactone hydrolysis in human liver microsomes, which may be mirrored by paraoxonase activity in other tissues [41]. Moreover, clinical studies find that PON1 polymorphisms influence statin response and paraoxonase activity is modulated by statin therapy, highlighting the physiological relevance of PON1‐mediated statin metabolism beyond the liver [43]. Thus, paraoxonase‐mediated hydrolysis plays an important role in the activation and pharmacodynamics of lactone‐form statins in diverse cellular environments. Beyond this core pharmacophore, structural variations, particularly in the ring systems appended to the moiety, further classify statins into type 1 and type 2 [44], where type 1 statins feature a decalin ring and type 2 possess larger, more hydrophobic ring structures [44, 45]. These modifications influence lipophilicity, membrane permeability and tissue distribution, ultimately affecting both therapeutic efficacy and the potential for off‐target effects [40, 46].

Multiple studies have investigated the efficacy of both type 1 and type 2 statins against BC cells, with a particular focus on the lipophilic statins, which include simvastatin, atorvastatin, lovastatin, fluvastatin and cerivastatin. Among these, simvastatin and atorvastatin (type 2 statins) have demonstrated notable efficacy in both preclinical and clinical contexts, as summarised in Table 1. In vitro data reveal that these statins exert dose‐dependent cytotoxic effects across various BC cell lines, including MCF‐7 (luminal A) and MDA‐MB‐231 (triple‐negative), at micromolar concentrations ranging from approximately 0.2–100 μM [51]. These effects manifest as inhibition of proliferation, induction of apoptosis and cell‐cycle arrest. Complementing these findings, in vivo studies using animal models and clinical trials employing clinically relevant doses of statins, resulting in plasma levels typically in the low nanomolar range (~0.02 μM), have reported reductions in tumour growth and increased apoptotic markers without significant toxicity [52]. For instance, a preoperative clinical trial administering high‐dose atorvastatin (80 mg/day for 2 weeks) demonstrated measurable biological activity by decreasing tumour proliferation in primary BC cases expressing HMG‐CoA reductase, as evidenced by shifts in tumour biomarkers [49]. This body of evidence highlights the therapeutic potential of simvastatin and atorvastatin against but also underscores the challenge posed by the disparity between the high concentrations required for in vitro efficacy and the lower, physiologically achievable concentrations in vivo. Addressing this gap will be essential for fully realising statins' anticancer applications.

**Table 1:** Summary of in vitro and in vivo studies assessing statin concentrations and effects in breast cancer models.

Breast cancer cell line (origin)	Statin(s) used	Concentrations tested (IC_50_ µM)	Model type (In vitro/In vivo)	Key findings	References
MCF‐7 (luminal A), MDA‐MB‐231 (TNBC)	Simvastatin, Atorvastatin	4–10	In vitro	Induced cell cycle arrest, apoptosis; differential sensitivity	[47, 48]
MCF‐7 (luminal A), T47D (luminal A)	Simvastatin	0.0001–0.05	In vitro and In vivo	Statins at clinical doses reduced tumour growth	[49]
Multiple breast cancer lines (e.g., MDA‐MB‐231)	Atorvastatin, Simvastatin	Atorvastatin (0.2–61) Simvastatin (0.2–50)	In vitro	Dose‐ and subtype‐dependent cell viability reduction	[50]
MCF‐7 (luminal A)	Simvastatin, Atorvastatin	Up to 100	In vitro	~70% proliferation inhibition at the highest dose	[51]
Breast cancer patients (various subtypes)	Simvastatin	Oral 0.02 (approximate plasma level)	In vivo (window‐of‐opportunity trial)	Increased markers of apoptosis and cell cycle arrest in tumour tissue posttreatment	[52]
Mouse models of breast cancer metastasis	Atorvastatin	Clinically relevant doses (~0.02 μM plasma)	In vivo	Suppressed metastatic proliferation without affecting primary tumour growth	[53]

Building on this translational approach, the MASTER trial (MAmmary cancer STatins in ER positive breast cancer) is a large, double‐blind, randomised, placebo‐controlled phase III study in Denmark evaluating whether adding high‐dose atorvastatin (80 mg/day for 2 years) to standard (neo)adjuvant therapy improves invasive disease‐free survival (IDFS) and overall survival (OS) in women with early‐stage, estrogen receptor (ER)‐positive BC [27, 54]. This trial represents a critical effort to bridge the gap between preclinical efficacy at high concentrations and clinically achievable statin exposure, and its outcome is expected to clarify whether statins can meaningfully improve BC outcomes when used as adjunctive therapy at standard dosing [27, 54].

BC can be broadly classified into molecular subtypes based on the presence or absence of hormone receptors and human epidermal growth factor receptor 2 (HER2) expression [55]. HER2 is a transmembrane tyrosine kinase receptor involved in regulating cell growth, survival and differentiation. Its overexpression or gene amplification occurs in approximately 15%–20% of BC and is associated with increased tumour aggressiveness [55]. Among the subtypes, hormone receptor‐positive BC, which expresses estrogen and/or progesterone receptors (ER and PR), is the most common and is typically associated with more favourable prognoses and responsiveness to endocrine therapies [56]. In contrast, TNBC, which lacks expression of ERs, PRs and HER2, is more aggressive and associated with poorer clinical outcomes [57]. MCF‐7 is a well‐established model of hormone receptor‐positive BC, while MDA‐MB‐231 represents the triple‐negative subtype [58, 59].

Several primary research studies have demonstrated that lipophilic statins, especially simvastatin (a type 1 statin), are effective at inhibiting the proliferation of BC cell lines such as MCF‐7 and MDA‐MB‐231 [47]. For example, Rezano et al. [47] showed that simvastatin exhibited a highly cytotoxic effect on both MCF‐7 and MDA‐MB‐231 cells, with half‐maximal inhibitory concentration (IC_50_) values of 8.9 μM for MCF‐7 and 4.5 μM for MDA‐MB‐231 after 48 h of treatment. The cytotoxic effect was dose‐dependent and time‐dependent, with increasing concentrations of simvastatin leading to greater cell death in both lines [47]. Additional studies confirm these findings: Shen et al. [48], Bai et al. [60] and Buranrat et al. [61] reported that simvastatin reduced MCF‐7 colony formation at concentrations ranging from 2.5 to 50 μM, and other groups observed significant inhibition of MDA‐MB‐231 proliferation at concentrations as low as 1–5 μM over 48 h. These results indicate that simvastatin is particularly effective against MDA‐MB‐231 cells at lower micromolar concentrations, while MCF‐7 cells require slightly higher doses for similar effects.

In contrast, studies investigating atorvastatin (a type 2 statin) found that while it can reduce the viability of MDA‐MB‐231 cells at 10 μM, MCF‐7 cells are relatively resistant to atorvastatin at this concentration, showing little or no reduction in cell viability [62]. This suggests that among the statins tested, simvastatin demonstrates the most consistent and potent cytotoxic activity against both MCF‐7 and MDA‐MB‐231 BC cell lines in vitro, with effective concentrations typically in the range of 4–10 μM depending on the cell type and assay conditions [47, 48, 60, 62]. For this reason, the section that follows will examine the impact of simvastatin on BC cell lines, with a focus on its pharmacological functions, mechanisms of action and its influence on molecular pathways related to lipid metabolism and cell signalling.

The concentrations of statins shown to induce cytotoxic effects in BC cell lines in vitro generally fall within the micromolar range, which substantially exceed plasma concentrations typically achieved in patients undergoing standard statin therapy. For instance, studies report that simvastatin exerts half‐maximal inhibitory effects (IC_50_) on MDA‐MB‐231 and MCF‐7 BC cells at concentrations between approximately 4 and 9 μM [47, 48]. In contrast, clinical pharmacokinetic data indicate that peak plasma levels of simvastatin in patients are within the low nanomolar range, typically less than 100 nM, which raises concerns regarding the physiological relevance of these in vitro findings. Moreover, administering statins at doses sufficient to achieve micromolar plasma concentrations may be clinically unfeasible due to well‐documented toxicities. Clinical pharmacokinetic studies indicate that plasma concentrations of commonly prescribed statins such as atorvastatin and simvastatin in patients receiving standard doses typically remain in the low nanomolar range, often below 50 ng/mL (approximately 0.1 μM) [63, 64]. Achieving micromolar plasma concentrations would require doses far exceeding those approved for clinical use, which significantly increases the risk of adverse effects.

High systemic exposure to statins is associated with adverse effects such as skeletal muscle myopathy and hepatotoxicity; for example, the risk of statin‐induced myopathy increases significantly at elevated doses [65]. Additionally, atorvastatin treatment has been associated with reductions in coenzyme Q10 levels, contributing to mitochondrial dysfunction and potentially to neurotoxic side effects [66]. These data suggest that the anticancer effects observed in vitro at micromolar statin concentrations may not translate directly into clinical efficacy without risking unacceptable toxicity. Therefore, careful consideration of dosing, delivery strategies and patient safety is warranted when interpreting the potential utility of statins as anticancer agents. Potential links between statin use and increased cancer risks are outlined below.

## 3 Potential Links Between Statin Use and Increased Cancer Risk

Some clinical studies and meta‐analyses have explored the potential link between statin use and an increased risk of certain cancers, although the evidence remains inconclusive and sometimes conflicting. A large meta‐analysis of 35 randomised controlled trials found that statin therapy did not significantly reduce or increase overall cancer incidence or mortality, including for BC, indicating a neutral effect of statins on cancer risk [67]. Similarly, a large cohort study assessing statin users compared with matched control drug users reported no significant increase or decrease in the risk of breast, lung or colorectal cancers, suggesting that statin use is unlikely to confer substantial cancer risk alterations over typical follow‐up periods [68].

However, isolated findings have raised some concerns. For example, one clinical trial reported an increased BC incidence with pravastatin use over 5 years (12 BC cases vs. 1 in placebo; *p* = 0.002) [69]. Other observational studies have occasionally suggested marginal increases in BC risk or mixed results depending on statin type, duration and population studied, though these have been subject to confounding factors. Furthermore, preclinical rodent studies have at times raised concerns about carcinogenic potential at certain doses, but such findings have not translated consistently to human populations [68].

Collectively, the current clinical evidence does not support a significant increase in cancer risk associated with statin use, though long‐term data remain limited. Continued monitoring in larger, longer‐duration clinical trials and real‐world studies is warranted to fully elucidate any potential cancer risks, particularly for specific statins or patient subgroups.

## 4 Repurposing Simvastatin With Mechanistic Insights Into Its Anticancer Effects in BC Cells Compared to Its Conventional Lipid‐Lowering Role

Simvastatin is primarily used to treat hypercholesterolaemia and dyslipidaemia [14, 70, 71, 72], which in turn lowers circulating low‐density lipoprotein cholesterol (LDL‐C) as discussed earlier [14]. Importantly, the mevalonate pathway is active not only in hepatocytes but also in all mammalian cells, including malignant breast cells, where it plays a vital role in cellular function [73]. This pathway generates critical isoprenoid intermediates, such as farnesyl pyrophosphate and geranylgeranyl pyrophosphate, which are required for membrane integrity, protein prenylation and intracellular signalling processes essential for cell growth, differentiation and survival [60, 74].

Simvastatin exerts its cholesterol‐lowering effects in hepatocytes primarily through inhibition of HMG‐CoA reductase and subsequent reduction in endogenous cholesterol synthesis as discussed earlier. In cancer cells, simvastatin disrupts the production of mevalonate pathway–derived intermediates that are essential for the prenylation of small GTPases, including members of the Ras and Rho families [74, 75]. Small GTPases are a large family of hydrolase enzymes that act as molecular switches, cycling between active (GTP‐bound) and inactive (GDP‐bound) states to regulate diverse cellular processes such as growth, survival and cytoskeletal organisation [74]. Ras proteins are a subfamily of small GTPases that play a central role in transmitting signals from cell surface receptors to intracellular pathways controlling proliferation and survival [75]. Rho GTPases, another subfamily, are key regulators of actin cytoskeleton dynamics and are critical for cell migration and invasion [74]. By blocking the prenylation and proper membrane localisation of these GTPases, simvastatin impairs signalling pathways that are fundamental for cancer cell proliferation, survival, migration and invasion [74, 75].

**Figure 3:**
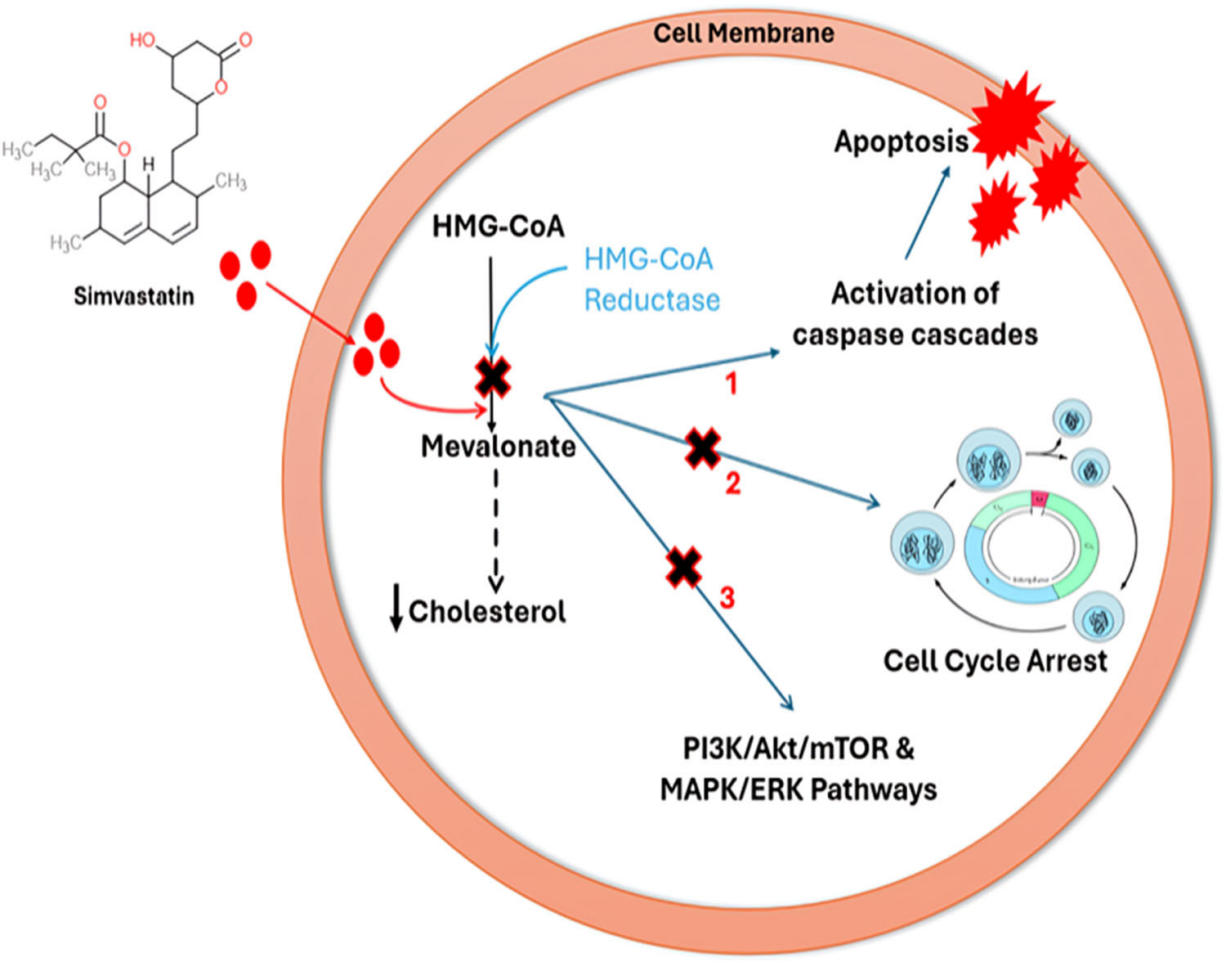
Illustrative diagram of simvastatin's mechanism of action in a breast cancer cell. Simvastatin inhibits the upregulated mevalonate pathway by blocking HMG‐CoA reductase, thereby reducing downstream metabolites essential for tumour growth and survival. This inhibition triggers three major anticancer effects: (1) induction of apoptosis via caspase activation and PARP cleavage; (2) cell‐cycle arrest through modulation of cyclins and CDK inhibitors; and (3) suppression of PI3K/Akt/mTOR and MAPK/ERK signalling pathways. Diagram created in PowerPoint. Information sourced from [60, 74, 75, 76].

In breast cells, both normal and malignant, the mevalonate pathway is crucial for the synthesis of these isoprenoid intermediates, supporting membrane structure and multiple signalling cascades [73]. In BC cells (Figure 3), this pathway is often upregulated, thereby contributing to tumour progression, metastasis and resistance to therapy [60, 77]. Simvastatin inhibits the mevalonate pathway in these cells by competitively blocking HMG‐CoA reductase, resulting in a reduction of downstream metabolites essential for cancer cell proliferation and survival [60, 74, 75]. This disruption triggers several anticancer mechanisms. First, simvastatin induces apoptosis, or programmed cell death, by activating caspase cascades, key enzymes involved in cellular dismantling. Laboratory studies have shown that simvastatin increases the activity of caspase‐3 and caspase‐7 and promotes the cleavage of poly (ADP‐ribose) polymerase (PARP), a hallmark of apoptosis [60, 75]. Second, simvastatin inhibits cancer cell proliferation by causing cell‐cycle arrest. It downregulates proteins that promote cell‐cycle progression, such as cyclin D1 and c‐myc, and upregulates inhibitors like p21 and p27, effectively halting the transition from the G1 to S phase of the cell cycle [60, 75]. Additionally, simvastatin suppresses two critical signalling pathways involved in cancer cell growth and survival: the PI3K/Akt/mTOR and MAPK/ERK pathways. It increases the expression of PTEN, a tumour suppressor and reduces the phosphorylation (activation) of key proteins in these pathways, thereby inhibiting their activity [75]. These effects collectively lead to reduced tumour cell proliferation and enhanced cell death. Importantly, the antitumour actions of simvastatin can be reversed by supplementing cells with mevalonic acid, which restores mevalonate or its downstream isoprenoid intermediates [60, 74, 75, 76].

Importantly, the same biochemical pathway underlies simvastatin's widely proven utility in cardiovascular disease management. For cholesterol lowering, simvastatin is typically prescribed at doses resulting in plasma concentrations in the low nanomolar range, approximately 0.01–0.02 μM [47]. In contrast, the half‐maximal inhibitory concentrations (IC_50_) of simvastatin for BC cell lines such as MCF‐7 and MDA‐MB‐231 are reported in the micromolar range, roughly 4.5–9 μM depending on the cell line and assay conditions [47, 48]. This indicates that the concentrations required for in vitro anticancer effects are approximately 200–900 times higher than those achieved with standard cholesterol‐lowering doses.

This substantial difference in concentration requirements highlights the pharmacokinetic challenge in repurposing simvastatin for oncology: while its cardiovascular benefits are achieved at low plasma levels with a well‐characterised safety profile, achieving anticancer efficacy may demand higher doses or alternative delivery methods to reach micromolar concentrations in tumour tissues without undue toxicity.

Resistance to simvastatin in BC further complicates its clinical translation. Cellular adaptations such as upregulation of the DNA replication licensing factor MCM7 and disruptions in retinoblastoma protein (Rb) signalling blunt simvastatin‐induced apoptosis and growth inhibition, particularly in tamoxifen‐resistant BC cells [78]. The pro‐inflammatory tumour microenvironment driven by the senescence‐associated secretory phenotype (SASP) promotes endocrine resistance [79]. SASP is characterised by irreversible cell‐cycle arrest in senescent cells accompanied by secretion of a mix of cytokines, chemokines, growth factors and proteases that remodel the tumour microenvironment and promote inflammation and therapy resistance [79]. Simvastatin may partially counteract SASP effects by inhibiting signalling pathways, including Rho GTPases, which are small G‐proteins crucial for regulating cytoskeletal dynamics, cell motility and SASP factor production [79]. However, compensatory cellular mechanisms limit simvastatin's efficacy. Moreover, pharmacokinetic barriers such as the low plasma concentrations achievable with conventional dosing and potential drug efflux mechanisms further challenge attaining therapeutic intracellular drug levels in tumour cells [60, 74, 75, 77].

Together, these resistance mechanisms and pharmacokinetic hurdles underscore the complexity of safely and effectively exploiting the mevalonate pathway dependencies in BC cells for therapeutic benefit. The mevalonate pathway produces essential metabolites like farnesyl pyrophosphate (FPP) and geranylgeranyl pyrophosphate (GGPP) that facilitate prenylation of proteins, including Rho GTPases, critical for oncogenic signalling, proliferation, survival and metastasis in BC. Simvastatin inhibits HMG‐CoA reductase, the rate‐limiting enzyme in this pathway, resulting in suppression of downstream oncogenic pathways such as PI3K/Akt/mTOR and MAPK/ERK, induction of apoptosis via intrinsic and JNK/CHOP/DR5 pathways and downregulation of metastasis‐associated proteins, including RHAMM, through transcriptional regulation by Hippo pathway effectors (YAP/TEAD). These actions collectively disrupt BC cell proliferation, survival, invasion and migration [74, 75, 77, 80].

Addressing pharmacokinetic challenges to achieve effective micromolar concentrations in tumour tissues, overcoming cellular resistance involving the tumour microenvironment and molecular adaptations and clarifying the therapeutic window are critical research priorities. This need is heightened by simvastatin's established clinical safety in cardiovascular disease via the same biochemical pathway, contrasting with the higher doses likely required for anticancer effects.

Overall, the presence and functional significance of the mevalonate pathway in BC cells underpin simvastatin's ability to induce apoptosis, arrest the cell cycle and inhibit oncogenic signalling, highlighting its potential as a therapeutic agent in oncology beyond its established role in cardiovascular disease. Given its well‐established use in managing cardiovascular disease via the same biochemical pathway, these findings underscore the value of comparing simvastatin's clinical applications in cancer treatment settings, as discussed below.

## 5 Exploring Simvastatin's Roles in Lipid Management and BC From Cholesterol to Oncology

Simvastatin has well‐established clinical applications in hypercholesterolaemia disease and is currently being investigated for its therapeutic potential in oncology, particularly in BC, as discussed earlier.

In contrast, simvastatin is being experimentally investigated for oncology applications, but its anticancer effects in vitro require much higher concentrations, typically in the micromolar range. For example, in BC cell lines, simvastatin inhibits cell proliferation with IC₅₀ values of 8.9 µM (8,900 nM) for MCF‐7 cells and 4.5 µM (4,500 nM) for MDA‐MB‐231 cells after 48 h of treatment [47], and similar results are observed in other studies (IC₅₀ = 7.98 µM for MDA‐MB‐231 after 72 h) [48].

Despite promising preclinical and early experimental data showing that simvastatin can inhibit BC cell proliferation and metastasis at micromolar concentrations, a significant gap remains in translating these findings to clinical benefit. Most in vitro studies demonstrate anticancer effects at concentrations (4.5–8.9 µM) much higher than those achieved with standard clinical dosing for lipid lowering, and robust clinical trials confirming improved cancer outcomes in BC patients are lacking [48]. While some observational and experimental studies suggest statins may reduce BC specific mortality or enhance chemosensitivity, the evidence is not definitive, and large, prospective randomised controlled trials are needed to establish efficacy, optimal dosing and safety for simvastatin as an adjunct or primary cancer therapy [81, 82]. This translational gap highlights the need for further research to clarify the role of simvastatin in oncology and to determine whether its preclinical antitumour effects can be realised in patients.

## 6 Conclusion

Statins exert anticancer effects through multiple mechanisms, including disruption of the mevalonate pathway, inhibition of cholesterol synthesis and interference with protein prenylation processes critical to cancer cell proliferation and metastasis. Among them, simvastatin, a lipophilic type 1 statin, has shown the most consistent antitumour activity in preclinical BC models by suppressing proliferation, inducing apoptosis and inhibiting invasion and migration. These findings highlight its potential beyond lipid‐lowering indications. However, despite promising preclinical results, clinical translation remains limited. Large‐scale, well‐designed trials are needed to evaluate simvastatin's therapeutic potential, optimal dosing and safety in BC patients.

## Author Contributions

**Rohina Alim:** conceptualization, writing – original draft, writing – review and editing, methodology. **H. M. Kasuni Akalanka:** conceptualization, writing – original draft, methodology, writing – review and editing.

## Ethics Statement

The authors have nothing to report.

## Consent

The authors have nothing to report.

## Conflicts of Interest

The authors declare no conflicts of interest.

## Data Availability

Data sharing is not applicable to this article as no data sets were generated or analysed during the current study.
